# Sensory Processing of Time and Space in Autistic Children

**DOI:** 10.3390/children12101366

**Published:** 2025-10-10

**Authors:** Franz Coelho, Belén Rando, Mariana Salgado, Ana Maria Abreu

**Affiliations:** 1 Universidade Católica Portuguesa, Center for Interdisciplinary Research in Health (CIIS), 1649-023 Lisbon, Portugal; 2 Universidade Católica Portuguesa, Faculty of Health Sciences and Nursing (FCSE), 1649-023 Lisbon, Portugal; marianasalgado1999@gmail.com; 3 Centre for Public Administration and Public Policies (CAPP), Institute of Social and Political Sciences, Universidade de Lisboa, 1300-666 Lisbon, Portugal; mcalvo@iscsp.ulisboa.pt; 4 Insight, Piaget Research Center for Ecological Human Development, 2805-059 Almada, Portugal

**Keywords:** Autism Spectrum Disorder, space, time, sensory processing

## Abstract

**Highlights:**

**What are the main findings?**

- Autistic children showed slower reaction times across visual temporal, auditory temporal, and visuospatial tasks, reflecting a speed processing deficit
- Despite slower responses, autistic children demonstrated higher accuracy in visual and auditory temporal tasks, indicating a strength in temporal integration

**What is the implication of the main finding?**

- Autism reflects a preference for accuracy over speed, underscoring the need for interventions that alleviate time pressure and promote structured, slower-paced environments

**Abstract:**

**Background/Objectives**: Autism is characterized by atypical sensory processing, which affects spatial and temporal perception. Here, we explore sensory processing in children with autism, focusing on visuospatial and temporal tasks across visual and auditory modalities. **Methods**: Ninety-two children aged 4 to 6 participated, divided into three groups: autism (*n* = 32), neurotypical chronological age-matched controls (*n* = 28), and neurotypical developmental age-matched controls (*n* = 32). The autism group consisted of high-functioning children (26 boys). The participants completed computer-based tasks requiring spatial and temporal processing. Response accuracy and reaction times were recorded. **Results**: The autism group demonstrated higher accuracy in temporal tasks (visual and auditory modalities) and comparable accuracy in visuospatial modality, but slower response times in all tasks compared to both neurotypical controls. These results suggest a strategy that prioritizes accuracy over speed, while preserving spatial and temporal processing in autism. **Conclusions**: These findings suggest that temporal processing, rather than the sensory modality, drives decision-making strategies in children with autism. Our findings highlight the need for interventions aligned with autistic children’s slower but accurate processing style to support social interaction and reduce stress. In a fast-paced digitalized world, autistic children might benefit from slower, balanced, and inclusive, evidence-based approaches that align with their cognitive rhythm and reduce overstimulation. By incorporating these unique strategies, targeted programs can enhance the quality of life and adaptive skills of children with autism, thereby fostering better integration into social and sensory-rich environments.

## 1. Introduction

In 1943, Leo Kanner identified autism as a neurodevelopmental condition characterized by challenges in social interaction and atypical behaviors [1]. Historically misdiagnosed as childhood schizophrenia [2], the understanding of autism has evolved significantly [3]. The DSM-5 now defines Autism Spectrum Disorder (ASD) as impairments in communication, social interaction, and restricted behaviors of varying severity [4]. Autism diagnoses initially focused on cognitive and social deficits [5]. Recent updates in the DSM-5 and ICD-11 incorporate sensory characteristics, improving diagnostic precision and recognizing the social impact of sensory responses [6,7,8].

### 1.1. Sensory Processing in Autism

Sensory processing refers to how individuals interpret and organize input from various sensory systems, including auditory, visual, gustatory (taste), olfactory (smell), somatosensory (touch and proprioception), vestibular (balance and spatial orientation), and interoceptive (internal bodily signals) [9]. When numerous highly variable sensory inputs are combined to interpret complex stimuli, it can disrupt later perception and hinder cognition—an effect that may particularly impact autistic individuals due to potential difficulties in processing complex sensory information [10]. Sensory processing differences distinguish Autism from other developmental disorders [11]. Individuals with autism face challenges in central coherence [12], visual processing [13], spatial perception [14], and motion and auditory processing [15,16]. These differences affect holistic visual interpretation and facial expression recognition [17,18,19]. Neurotypical children with sensory integration deficits also struggle with daily activities, highlighting the need for early screenings [20]. In autism, low-level sensory deficits, such as motion perception challenges, are linked to high-level social deficits [21,22,23]. Global processing atypicalities hinder sensory integration [24]. Autism-related sensory deficits, such as fragmented visual processing [25] and hypersensitivity to sounds, further complicate social cue interpretation [26,27,28,29].

### 1.2. Spatial and Temporal Perception Modalities Challenges

Sensory processing refers to the neurobiological handling and organization of sensory input from the body and environment, which is later integrated into higher-level cognitive functions such as perception—a holistic inference process operating across representational levels to interpret experience [30,31]. Spatial and temporal dimensions are essential to the cognitive processes underlying perception, shaping our conscious experience of the surrounding world through sensory processing [32,33]. A study suggests that the autistic cortex undergoes specific, low-level alterations in neural circuits responsible for perceptual processing, including primary sensory regions [34]. Autistic individuals show difficulties integrating spatial and temporal information [35]. Spatial awareness aids navigation, while temporal understanding supports organizing actions [36,37], emerging as vital cognitive components for daily life. Research suggests that people with autism have a weakness in recalling specific events, including their details and the broader spatiotemporal context, affecting the ability to mentally reconstruct the spatial setting, a capacity known as scene construction [38].

Temporal deficits involve challenges in perceiving time passage and event duration [39], often manifesting across sensory modalities [40]. These difficulties disrupt the integration of time intervals [41] and time estimation abilities [42]. Temporal duration perception is essential for sequencing events and coordinating activities, such as motor actions or conversations [7,43,44,45]. Consequently, daily activities, social interactions, and motor skills are adversely affected [46]. Temporal processing, critical for interpreting sensory stimuli and events over time, is often impaired in autism [47,48]. These deficits affect auditory information integration and speech perception, especially in complex settings [29,49]. An event-related potential (ERP) study reveals that autistic children exhibit reduced neural amplitudes and delayed latencies in response to temporal variations [40]. Such challenges hinder comprehension of event sequences and spatial relationships [50,51].

Neurotypical children develop time perception gradually, recognizing sequences and durations by age 5 [52,53]. Conversely, autistic children struggle with time estimation, sequencing, and spatial organization [36,50]. Deficits in integrating spatial and temporal information exacerbate difficulties in social and motor tasks [46].

### 1.3. Sensory Processing Through Spatial-Temporal Modalities

Sensory deficits in autism arise from distinct origins, with spatial integration challenges differing from auditory issues linked to temporal deficits [35,50]. These auditory deficits disrupt social interaction and communication, indicating that spatial difficulties alone cannot fully explain these impairments [7,27]. Temporal integration, essential for speech comprehension, along with pattern recognition, divided attention across multiple sensory cues, and social signal interpretation, compounds these challenges [54,55,56,57]. Auditory deficits in autism include challenges in integrating sound duration and rhythm, along with difficulties in intensity and directionality [58,59,60]. These challenges are exacerbated in social environments with overlapping and repetitive auditory stimuli, where sensory overload and auditory filtering issues prevail [61]. Sensory deficits can be related to spatial and temporal challenges, complicating how individuals with autism interact with their environment [62,63].

Understanding sensory domain interplay is vital for addressing integration issues in autism [64]. Autistic individuals often struggle to modulate and integrate sensory input effectively, which highlights the need to develop interventions involving sensory integration, designed to help organize sensory information for functional use [9,65]. Synchronization difficulties constitute a hallmark of autism, impacting motor coordination, communication, and behavior [66,67,68,69], with temporal coordination being crucial for interpersonal interactions [70]. Temporal processing deficits disrupt event prediction, sequence organization, and social adaptation, leading to frustration and challenges in interpreting social nuances [25].

### 1.4. Importance of Early Identification and Intervention

Information integration difficulties are very relevant in autism [71]. These challenges hinder the combination of sensory, emotional, and cognitive stimuli, complicating behavior, communication, and social interaction [72,73]. Early identification of sensory challenges is important to prevent these issues from escalating, improve developmental outcomes, and enhance the quality of life for both children and their families [74,75,76]. Early intervention strengthens communicative, social, and cognitive competencies [77].

During early childhood, heightened brain plasticity allows significant adaptation to new stimuli and experiences [78,79]. These experiences include a range of interactions that stimulate essential areas for sensory, motor, cognitive, and social skills development [80]. Physical contact is also crucial for emotional bonding [81]. Interactions such as sensory play and peer activities promote cognitive and social skill development [82,83]. Leveraging this plasticity through early intervention reduces social isolation and enhances adaptability [84,85], supporting gains in language, play, and social communication. [86], and mitigating comorbidities related to mental health, such as anxiety and depression [87,88].

### 1.5. Research Objectives and Hypothesis

Individuals with autism exhibit behavioral atypicalities linked to temporal processing deficits [28,89]. These deficits underlie higher-order impairments [40] and difficulties in sensory integration, like spatial and temporal domains [90,91]. Challenges in processing quantities manifest in both visual and auditory modalities [92], indicating a common origin affecting integration and social interaction [61,93]. Therefore, this study aims to examine sensory processing domains that pose challenges for autistic children—specifically, visual and auditory—while also addressing the temporal and spatial aspects of perception, as these higher-order dimensions are likewise areas of difficulty for this population. This study aims to examine how children with autism, compared with neurotypical children, process temporal visual space (duration of one visual item relative to another), temporal auditory space (duration of auditory items in relation to one another), and physical visual space (distance between visual stimuli), considering the accuracy and speed tradeoff. To our knowledge, this is one of the first studies to examine sensory processing across visual and auditory modalities while considering spatial and temporal perception domains, using dual control groups to enhance the comparative validity of the research. Table 1 presents our hypotheses.

To better understand the nature of sensory processing through spatial-temporal modalities in autism, we proposed four possible interpretative pathways based on these hypotheses presented in Table 1. First, if difficulties appear in both visual and auditory temporal modalities but not in visuospatial processing, this suggests that autistic deficits are not tied to a specific sensory modality but instead reflect a broader challenge in temporal integration. Second, if impairments are found in visual, temporal, and visuospatial domains, but not in the auditory modality, it may indicate that difficulties are primarily related to visual information processing. Third, if deficits are observed across all three areas—visual and auditory temporal, and visuospatial—this would point to a more generalized challenge in integrating spatial, temporal, and physical information across sensory modalities. Fourth, if, rather than difficulties, autistic children demonstrate strengths or enhanced performance in any of the three domains—visual temporal, auditory temporal, or visuospatial—this would suggest not deficits, but potential advantages in sensory processing and spatial-temporal perception.

## 2. Materials and Methods

### 2.1. Participants

Considering that typical development is asynchronous and individualized, and autism follows distinct, non-linear paths, age-matched controls enable clearer comparisons of age-specific features and avoid assuming uniform developmental progression, clarifying how autism traits emerge or differ across maturation stages [94]. Nonetheless, acknowledging that autistic individuals often show distinct brain development patterns—shaping behavior, neural activity, and structure over time—comparing them to developmentally matched peers is also crucial, as merging age ranges or using age merely as a control can obscure meaningful group differences [95]. Thus, aligning with prior research designs that compare autistic children to both chronological and developmental peers [96], this study used two distinct control groups (CG)—one chronologically matched and one developmentally matched—to more accurately identify cognitive differences in comparison to autistic children. Only high-functioning children with autism were included in the study, as this was necessary to ensure that participants were able to complete the cognitive assessments. All children had normal or corrected-to-normal vision at the time of testing; however, specific information on the use of glasses or corrective lenses was not systematically collected.

An a priori power analysis was computed using G*Power v.3.1.9.4 and F tests for MANOVA (Repeated measures, within-between interaction) [97]. We considered a medium effect size (f(V) = 0.35), a power of 0.80, and an alpha of 0.05 for three groups and nine response variables, yielding a sample size *n* = 86. Considering the sample size determined by the power analysis and the possibility of losing participants during the experiment, a total of *n* = 92 individuals were recruited for the study. Children in this study were recruited from two local school clusters (neurotypical children as CG) and a Child Development and Support Center (clinical autistic group—AG). Data collection for the AG occurred in a controlled setting with a multidisciplinary team to ensure credibility, participant well-being, and immediate support if needed. The sample included 60 neurotypical children and 32 children with autism, aged 4 to 6 years, matched by chronological and developmental age (see Table 2). Raven’s Coloured Progressive Matrices [98,99], a 25–30-min non-verbal test assessing logical and abstract reasoning, ensured developmental matching. Given the challenges of autistic people (such as difficulties in communication) that complicate the use of standardized intelligence tests, there is no universally reliable index of cognitive functioning in autistic children, though Raven’s Coloured Progressive Matrices may offer a suitable and valid alternative [100,101]. The Raven’s Coloured Progressive Matrices is a non-verbal reasoning test designed to minimize cultural and scholastic bias, and it demonstrates excellent psychometric properties in the Portuguese population, with high internal consistency (Cronbach’s α = 0.94) and strong test–retest reliability (r = 0.82, *p* < 0.001) [102]. The commonly used Wechsler Intelligence Scale for Children (WISC) often underestimates intelligence in autistic children, whereas Raven’s Coloured Progressive Matrices offers a more suitable alternative, better representing autistic intelligence [102,103]. Therefore, the developmentally matched CG in this study was based on comparable scores on Raven’s Coloured Progressive Matrices. Administered in a calm setting before the intervention, this test involved completing geometric patterns. All children in the autism group had a prior clinical diagnosis of autism made by qualified professionals according to DSM-5 through external specialized services. To ensure consistency across participants from different referral pathways, the Childhood Autism Spectrum Test (CAST) [104] was additionally applied as a standardized confirmation measure. CAST is a 10-min parental checklist identifying autism traits. To our knowledge, there are no European Portuguese adaptations of the CAST to date. Therefore, given the cultural and linguistic similarities between Spanish and Portuguese, we considered the Spanish version for data validity, which has shown strong psychometric properties with high internal consistency (Cronbach’s α = 0.826), good convergent validity with Autism Diagnostic Interview Revised (ADI-R) and Autism Diagnostic Observation Schedule—Second Edition (ADOS-2), and proper screening performance (sensitivity = 83.9%, specificity = 92.5%) [105]. CAST was applied the same day as Raven’s Coloured Progressive Matrices to streamline the process.

Inclusion criteria for the AG were: (i) ages 5–6, (ii) confirmed autism diagnosis, (iii) normal hearing, (iv) normal or corrected vision, and (v) no motor impairments. For the CG, the criteria were identical but excluded developmental disorders. Developmentally matched controls were paired with the AG using Raven’s Coloured Progressive Matrices. In accordance with the inclusion criteria, two participants from the AG under the age of 5 were excluded.

### 2.2. Procedure

In the initial phase, parents completed a 15-item checklist in 5 min to confirm the autism diagnosis, along with informed consent forms. Raven’s Coloured Progressive Matrices [98,99], a non-verbal test assessing logical reasoning through geometric pattern completion, was administered in a calm, controlled setting before the experiment, lasting 25–30 min. A sociodemographic questionnaire gathered participant details such as age, education, diagnosis, siblings, daycare attendance, and involvement with professionals, compiling clinical histories and ensuring inclusion criteria were met. This study received approval from the local Ethics Committee.

Customizable software, such as Cedrus SuperLab v.6.0, has been used to design computer-based tasks for assessing cognitive abilities in autistic individuals [106,107]. Compared to face-to-face settings, digital interaction may be less socially and cognitively overwhelming for autistic people due to reduced social demands, making the experience more comfortable and less stressful [108,109]. In line with this, our study used computer-based tasks to evaluate sensory processing, specifically the visual and auditory ones, during spatial and temporal perceptual cognitive processes in autistic children, aiming to provide a controlled, consistent, and socially low-pressure environment for assessment. Digital technologies are widely accessible, capable of capturing autism-relevant phenotypes, and feasible to administer in children’s everyday settings by non-specialists [110]. While standardized observational methods remain fundamental, they are constrained by subjectivity, limited precision, reliance on highly trained professionals, and scalability issues; digital behavioral assessments provide a promising pathway to enhance the identification, diagnosis, and monitoring of autism and other neurodevelopmental conditions [111]. Criterion-related validity reflects correspondence with established benchmarks, whereas reliability concerns the stability of measurements across repeated administrations [112]. Accordingly, computer-based tasks have been employed to examine sensory processing through customized paradigms created with Cedrus SuperLab, a methodology already used in prior research with autistic children [113,114], particularly emphasizing visual [115,116] and auditory [117,118] computerized stimuli, thereby supporting the collection of criterion-related valid and reliable data due to repeated conditions.

Experimental tasks were conducted using Cedrus SuperLab 6 on a laptop, measuring response accuracy and reaction times. Sessions lasted 20–25 min in quiet, low-noise environments at the clinic or schools, with participants seated in front of the laptop. An experimenter provided instructions and clarified doubts. The experimenter provided support in all groups and was already familiar with the children, which helped ensure a comfortable environment during task execution. The parents were not present during the experiments. All sessions took place in the same room with minimal distractions and controlled lighting. Tasks began with a detailed video and a pilot session, including three practice trials per task with alternate stimuli. The formal tasks—temporal visual processing, temporal auditory processing, and visuospatial processing—were implemented via Cedrus SuperLab 6 to ensure consistency and precision.

#### 2.2.1. Processing Tasks

Participants completed tasks assessing temporal visual, auditory, and visuospatial processing. Each trial began with a 500 ms black screen displaying a fixation cross, followed by a stimulus. After a 3000 ms interval, the same stimulus reappeared with altered properties. Participants responded to a question using a portable mouse, selecting “YES” (green button) or “NO” (red button). In all tasks, accuracy (proportion of correct responses) and mean reaction times (for correct and incorrect responses) were recorded as dependent variables. Trials were randomized, and each task included multiple repetitions.

#### 2.2.2. Visual Temporal Processing Task

**Figure 1:**
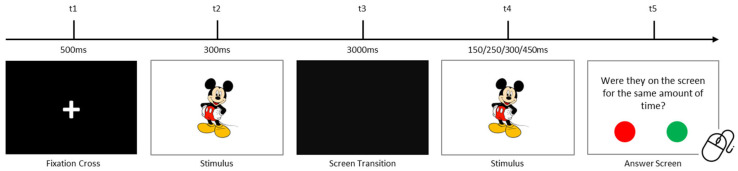
Temporal visual processing task procedure.

**Figure 2:**
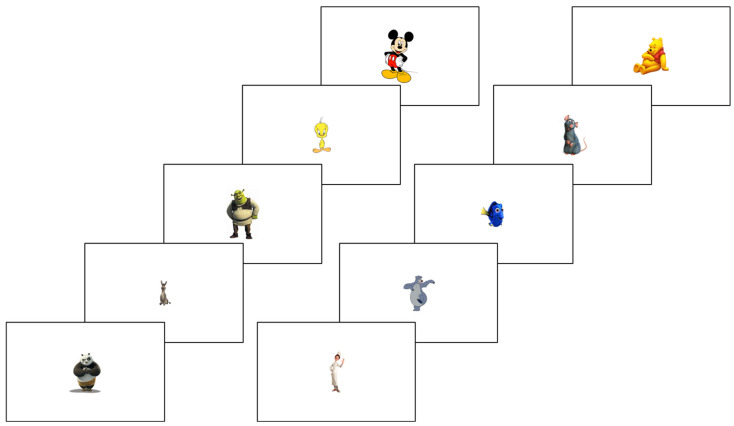
Temporal visual processing task stimuli.

This task assessed visual sensory processing by evaluating temporal perception. The stimulus was a 10 cm visual image (e.g., Mickey Mouse) initially displayed for 300 ms. Upon reappearance, its duration varied (150 ms, 250 ms, 300 ms, or 450 ms). Participants compared the two durations across 40 trials. Figure 1 illustrates the procedure. All the used stimuli are presented in Figure 2.

#### 2.2.3. Auditory Temporal Processing Task

**Figure 3:**
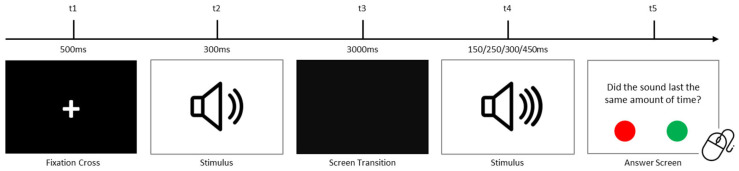
Temporal auditory processing task procedure.

This task assessed auditory sensory processing by evaluating temporal perception. This task mirrored the visual task but used auditory stimuli (tones). Durations matched those in the visual task, and 40 randomized trials were conducted. Figure 3 represents the auditory task procedure. All the stimuli are presented in the Open Science Framework (OSF) repository [119].

#### 2.2.4. Visuospatial Processing Task

**Figure 4:**
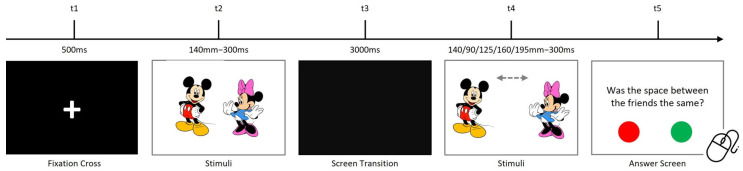
Visuospatial processing task procedure.

**Figure 5:**
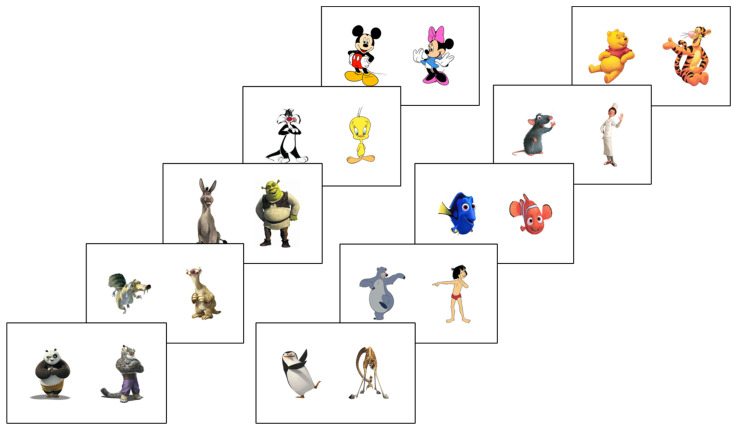
Visuospatial processing task stimuli.

This task assessed visual sensory processing by evaluating spatial perception. Participants compared the distances between two 10 cm images. Initially separated by 140 mm, the reappearance distances varied (90 mm, 120.5 mm, 140 mm, 160 mm, or 190.5 mm). Each image pair was displayed for 500 ms, with 50 randomized trials presented. Figure 4 details the procedure. All the used stimuli are presented in Figure 5.

### 2.3. Data Recording and Analysis and Community Involvement

The protocol used Cedrus SuperLab 6 software to record responses and reaction times, with stimuli presented on a 1920 × 1200 resolution laptop. Data were analyzed in IBM Statistical Package for the Social Sciences (SPSS) 29 to examine group differences and deficit profiles. During the pilot phase, the experimenter ensured participant comfort, allowing adjustments and gathering feedback to refine sessions. After publication, in line with the journal’s copyright, findings will be shared with autistic individuals, families, community providers, policymakers, and schools to improve educational and social conditions.

## 3. Results

A factorial repeated measures MANOVA (A × B) was conducted with Group (AG, Chronological CG, Developmental CG) as the between-subjects variable and Sensory Modality (visual, auditory, visuospatial) as the within-subject variable. The nine dependent variables were: i. Proportion of correct responses: visual temporal, auditory temporal, and visuospatial processing; ii. Mean reaction times (correct and incorrect responses): visual temporal, auditory temporal, and visuospatial processing.

Regarding assumptions, the independence of observations among participants was assumed. Multivariate outliers were identified via Mahalanobis distances (critical χ^2^ = 27.88, α = 0.001) and Leverage measures (<0.2). Five cases exceeding the Mahalanobis threshold were excluded (three from AG, two from Chronological CG) [120], leaving final sample sizes of *n* = 29 (AG), *n* = 28 (Developmental CG), and *n* = 30 (Chronological CG).

Shapiro-Wilks tests indicated non-normality for most variables. However, MANOVA is robust to non-normality caused by skewness rather than outliers [121]. With respect to multicollinearity, high correlations between mean reaction times for correct and incorrect responses were found. For this reason, analyses were performed with and without mean reaction times for incorrect responses. Since results were consistent, all nine variables were retained. Scatterplots indicated linearity among some variables, though this was less evident in others. However, the absence of linearity is not expected to increase Type I error rates [121].

Box’s M test assessed the homogeneity of variance-covariance matrices across groups and was significant [Box’s M = 504.439; F(90, 19212.884) = 4.742; *p* < 0.001]. This may have been due to its sensitivity to non-normality, or to sample sizes [122].

Sphericity for Sensory Modality was validated for the proportion of correct responses and mean reaction times for incorrect responses, but not for mean reaction times of correct responses. Greenhouse-Geisser corrections were applied where sphericity was violated [Proportion of correct responses: W = 0.953, χ^2^ = 3.998, df = 2, *p* = 0.135; Mean reaction times for correct responses: W = 0.915, χ^2^ = 7.343, df = 2, *p* = 0.025; Mean reaction times for incorrect responses: W = 0.981, χ^2^ = 1.559, df = 2, *p* = 0.459].

The homogeneity of variances for the between-subjects factor (Group) was validated only for correct response proportions in auditory and visuospatial temporal processing. Results were as follows for respective response proportions: Visual temporal (correct): F(2,84) = 14.887, *p* < 0.001; Auditory temporal (correct): F(2,84) = 1.293, *p* = 0.280; and Visuospatial temporal (correct): F(2,84) = 1.811, *p* = 0.170. Results were as follows for mean reaction times: Visual (correct): F(2,84) = 40.837, *p* < 0.001; Auditory (correct): F(2,84) = 53.183, *p* < 0.001; Visuospatial (correct): F(2,84) = 50.251, *p* < 0.001; Visual (incorrect): F(2,84) = 31.903, *p* < 0.001; Auditory (incorrect): F(2,84) = 48.947, *p* < 0.001; and Visuospatial (incorrect): F(2,84) = 33.091, *p* < 0.001.

Regarding descriptive statistics of the full experimental design, Table 3 shows respective means and standard deviations.

The multivariate tests of the factorial MANOVA A × B with repeated measures were significant for the Group factor (Pillai’s Trace = 0.608, F(6, 166) = 12.075, *p* < 0.001, partial η^2^ = 0.304; Wilks’ Lambda = 0.422, F(6, 164) = 14.724, *p* < 0.001, partial η^2^ = 0.350; Hotelling’s Trace = 1.296, F(6, 162) = 17.501, *p* < 0.001, partial η^2^ = 0.393; Roy’s Largest Root = 1.239, F(3, 83) = 34.278, *p* < 0.001, partial η^2^ = 0.553), for the Sensory Modality factor (Pillai’s Trace = 0.880, F(6, 79) = 96.680, *p* < 0.001, partial η^2^ = 0.880; Wilks’ Lambda = 0.120, F(6, 79) = 96.680, *p* < 0.001, partial η^2^ = 0.880; Hotelling’s Trace = 7.343, F(6, 79) = 96.680, *p* < 0.001, partial η^2^ = 0.880; Roy’s Largest Root = 7.343, F(6, 79) = 96.680, *p* < 0.001, partial η^2^ = 0.880), and for the Group × Sensory Modality Interaction (Pillai’s Trace = 0.716, F(12, 160) = 7.433, *p* < 0.001, partial η^2^ = 0.358; Wilks’ Lambda = 0.350, F(12, 158) = 9.086, *p* < 0.001, partial η^2^ = 0.408; Hotelling’s Trace = 1.668, F(12, 156) = 10.842, *p* < 0.001, partial η^2^ = 0.455; Roy’s Largest Root = 1.546, F(6, 80) = 20.616, *p* < 0.001, partial η^2^ = 0.607).

**Figure 6:**
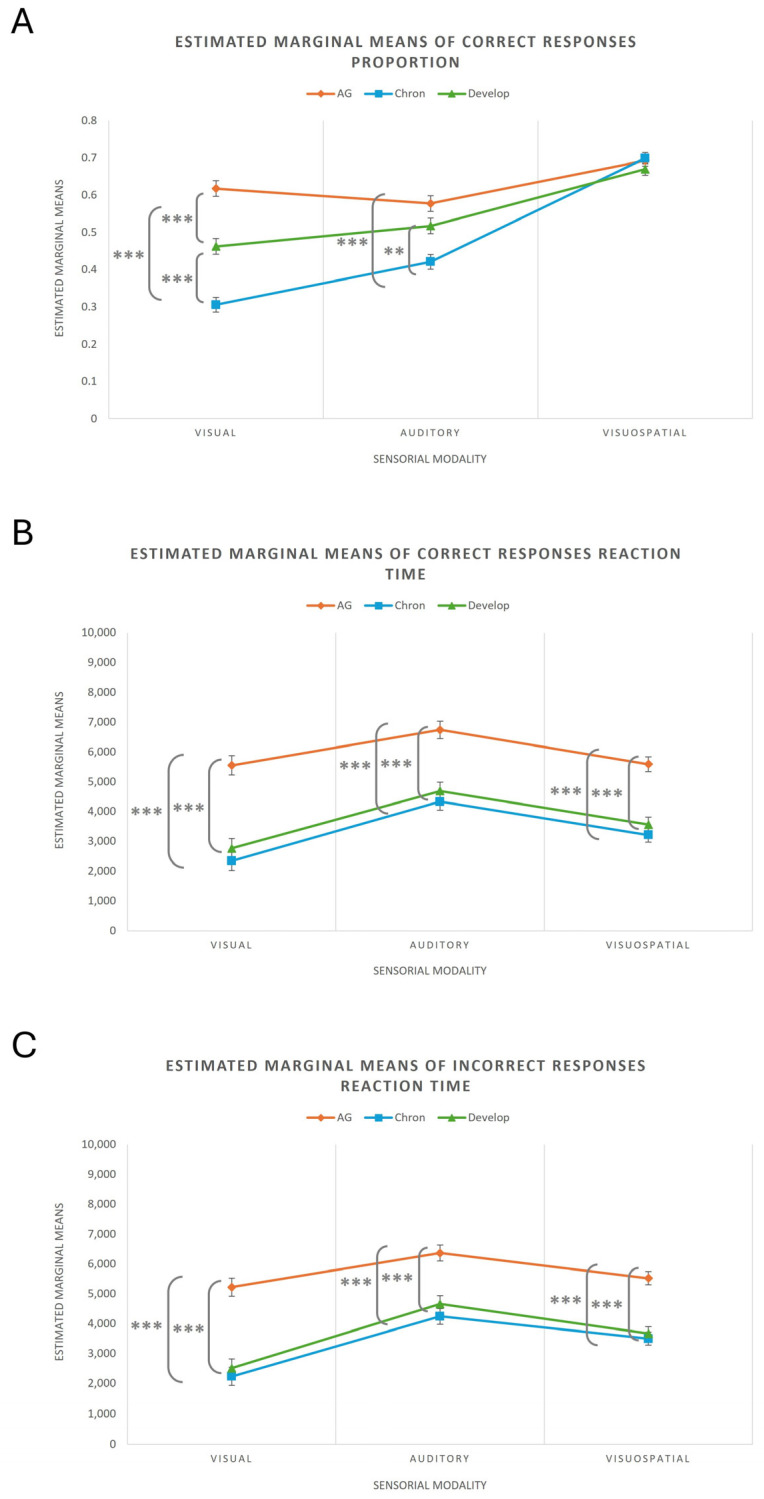
Effect of Interaction. Note. (**A**) Effect of Interaction on the Proportion of Correct Responses; (**B**) Effect of Interaction on Mean Reaction Time for Correct Responses; (**C**) Effect of Interaction on Mean Reaction Time for Incorrect Responses. Error bars are represented. ** *p* < 0.01; *** *p* < 0.001.

The univariate tests for the within-subject factor revealed significant main effects of Sensory Modality, indicating statistically significant differences across the dependent variables according to sensory modality. Likewise, significant interaction effects were observed between Sensory Modality and Group (Table 4). Figure 6 illustrates the interaction effects on the proportion of correct responses, mean reaction times for correct responses, and mean reaction times for incorrect responses.

The univariate ANOVA tests for the between-subjects factor showed significant main effects of the Group variable, indicating statistically significant differences in response variables according to the group (Table 5).

Since all effects in the design were significant, interpreting the results required: i. Estimating marginal means for the Group factor (between-subjects) and conducting multiple comparisons to analyze the main effects of the Group factor (see Table 6 for estimated marginal means); ii. Estimating marginal means for the Sensory Modality factor (within-subjects) and conducting multiple comparisons to analyze the main effects of the Sensory Modality factor (see Table 7 for estimated marginal means); iii. Decomposing the factorial design into simple designs, estimating simple means, and conducting multiple comparisons to analyze the simple effects of the Group factor while keeping the Sensory Modality constant (see Table 8 for estimated simple means).

Regarding the Group factor, the Games-Howell multiple comparison tests revealed statistically significant differences between the AG, Chronological CG, and Developmental CG in the proportion of correct responses and mean reaction times. For the proportion of correct responses, significant differences were observed between AG and Chronological CG (95% CI [0.114, 0.195], *p* < 0.001), AG and Developmental groups (95% CI [0.027, 0.133], *p* = 0.002), and Chronological and Developmental groups (95% CI [−0.128, −0.021], *p* = 0.004). On average, the AG exhibited a higher proportion of correct responses, followed by the Developmental group, and finally, the Chronological CG. Regarding the mean reaction time for correct responses, significant differences were observed between AG and Chronological CG (95% CI [1584.62, 3740.67], *p* < 0.001), AG and Developmental groups (95% CI [1170.11, 3402.79], *p* < 0.001), and Chronological and Developmental groups (95% CI [−726.16, −26.23], *p* = 0.033). The AG demonstrated significantly longer mean reaction times for correct responses, followed by the Developmental group, with the Chronological CG displaying the shortest mean reaction times. For the mean reaction time for incorrect responses, the AG showed significantly longer mean reaction times compared to both CG (AG vs. Chronological: 95% CI [1390.12, 3379.07], *p* < 0.001; AG vs. Developmental: 95% CI [1069.74, 3122.27], *p* < 0.001), while no statistically significant differences were observed between the Chronological and Developmental groups (95% CI [−621.94, 44.76], *p* = 0.101).

With respect to the Sensory Modality factor, the multiple comparison tests using Bonferroni correction revealed statistically significant differences across all comparisons. The proportion of correct responses was, on average, highest in the visuospatial modality, followed by the auditory modality, and lowest in the visual modality (Visual vs. Visuospatial: 95% CI [−0.258, −0.193], *p* < 0.001; Visual vs. Auditory: 95% CI [−0.070, −0.016], *p* < 0.001; Visuospatial vs. Auditory: 95% CI [0.150, 0.214], *p* < 0.001). Regarding the mean reaction time for correct responses, the auditory modality had the longest times, followed by the visuospatial modality, and finally the visual modality (Visual vs. Visuospatial: 95% CI [−859.52, −284.37], *p* < 0.001; Visual vs. Auditory: 95% CI [−2033.76, −1368.47], *p* < 0.001; Visuospatial vs. Auditory: 95% CI [−1388.90, −869.45], *p* < 0.001). For the mean reaction time of incorrect responses, the same pattern was observed (Visual vs. Visuospatial: 95% CI [−1208.04, −613.78], *p* < 0.001; Visual vs. Auditory: 95% CI [−2035.38, −1507.76], *p* < 0.001; Visuospatial vs. Auditory: 95% CI [−1143.53, −577.79], *p* < 0.001).

To interpret the interaction effects, the factorial design was decomposed into simple designs. This approach analyzed Group means while holding Sensory Modality constant, given their interdependence. Multiple comparisons of simple means were performed using Bonferroni adjustments.

When keeping the Sensory Modality constant, starting with the visual modality, significant differences were observed between the AG, Chronological, and Developmental groups in the average proportion of correct responses, with higher values in the AG, followed by the Developmental group, and lastly the Chronological CG (AG vs. Chronological: 95% CI [0.242, 0.383], *p* < 0.001; AG vs. Developmental: 95% CI [0.084, 0.227], *p* < 0.001; Chronological vs. Developmental: 95% CI [−0.228, −0.085], *p* < 0.001). Regarding the mean reaction time for correct responses, the AG again showed higher averages compared to the CG, with no statistically significant differences between the latter (AG vs. Chronological: 95% CI [2096.64, 4317.20], *p* < 0.001; AG vs. Developmental: 95% CI [1657.97, 3917.17], *p* < 0.001; Chronological vs. Developmental: 95% CI [−1539.66, 700.97], *p* = 1.000). The same pattern of results was observed for the mean reaction time for incorrect responses (AG vs. Chronological: 95% CI [1949.77, 4042.39], *p* < 0.001; AG vs. Developmental: 95% CI [1657.22, 3786.26], *p* < 0.001; Chronological vs. Developmental: 95% CI [−1330.11, 781.43], *p* = 1.000).

For the auditory modality, no significant differences were found between the AG and the Developmental group in the average proportion of correct responses, but the other comparisons were significant, with higher averages in the first two groups compared to the Chronological CG (AG vs. Chronological: 95% CI [0.085, 0.228], *p* < 0.001; AG vs. Developmental: 95% CI [−0.012, 0.133], *p* = 0.134; Chronological vs. Developmental: 95% CI [−0.168, −0.024], *p* = 0.005). Regarding the mean reaction time for correct responses, the AG again had higher averages compared to the CG, with no significant differences between the latter (AG vs. Chronological: 95% CI [1401.90, 3409.22], *p* < 0.001; AG vs. Developmental: 95% CI [1025.14, 3067.40], *p* < 0.001; Chronological vs. Developmental: 95% CI [−1372.03, 653.44], *p* = 1.000). The same pattern of results was observed for the mean reaction time for incorrect responses (AG vs. Chronological: 95% CI [1195.42, 3061.44], *p* < 0.001; AG vs. Developmental: 95% CI [762.36, 2660.86], *p* < 0.001; Chronological vs. Developmental: 95% CI [−1358.27, 524.62], *p* = 0.848).

For the visuospatial modality, there were no statistically significant differences between the groups in the average proportion of correct responses (AG vs. Chronological: 95% CI [−0.059, 0.046], *p* = 1.000; AG vs. Developmental: 95% CI [−0.030, 0.076], *p* = 0.894; Chronological vs. Developmental: 95% CI [−0.024, 0.082], *p* = 0.544). However, for the mean reaction time for correct responses, the AG once again had higher averages compared to the CG, with no significant differences between the latter (AG vs. Chronological: 95% CI [1533.07, 3217.84], *p* < 0.001; AG vs. Developmental: 95% CI [1168.46, 2882.55], *p* < 0.001; Chronological vs. Developmental: 95% CI [−1199.95, 500.05], *p* = 0.952). The same pattern of results was observed for the mean reaction time for incorrect responses (AG vs. Chronological: 95% CI [1267.49, 2791.05], *p* < 0.001; AG vs. Developmental: 95% CI [1079.63, 2629.70], *p* < 0.001; Chronological vs. Developmental: 95% CI [−943.26, 594.061], *p* = 1.000).

### Summary of Hypotheses and Findings

H1a, which proposed that autistic children show deficits in visual temporal processing compared to CG, regarding processing accuracy, was surprisingly not supported, as results revealed the opposite pattern: the AG demonstrated higher accuracy compared to both CG. Nonetheless, H1b, which proposed that autistic children show deficits in visual temporal processing compared to CG, regarding processing speed, was supported, as the AG demonstrated longer mean reaction times for both correct and incorrect responses compared to both CG.

H2a, which predicted auditory temporal processing deficits in autistic individuals, concerning processing accuracy, was also not supported, and the results showed the opposite. Indeed, the AG showed higher accuracy than the chronologically matched CG. However, H2b, which suggested that autistic children show deficits in auditory temporal processing compared to CG, related to processing speed, was confirmed, as the AG demonstrated slower mean reaction times for both correct and incorrect responses compared to both CG.

H3a, suggesting deficits in visuospatial processing for accuracy, was not supported, as there were no significant differences between the groups. On the other hand, H3b, related to deficits in visuospatial processing for speed, was confirmed, with the AG exhibiting longer mean reaction times for both correct and incorrect responses relative to both the CG.

Contrary to our expectation and consistent with the findings of H1a and H2a, our results contradict our original pathway derived from the hypotheses, which posited that autistic deficits could appear in visual and auditory temporal modalities but not in the visuospatial modality—as the AG demonstrated higher accuracy than both CG in visual temporal tasks and outperformed the chronologically matched CG in auditory temporal tasks—suggesting a strength in temporal integration related to accuracy in the autistic children. Nonetheless, in line with one of our expectations concerning the three previous hypotheses confirmation (H1b, H2b, and H3b), there were deficits across all three domains in speed processing—visual and auditory temporal, and visuospatial—as AG demonstrated slower mean reaction times for both correct and incorrect responses relative to both CG. This shows that autism challenges likely involve integrating spatial, temporal, and physical information across modalities concerning speed processing.

## 4. Discussion

This study investigated sensory processing in autistic children through visuospatial and temporal tasks across visual and auditory modalities, compared to chronological and developmental CG. Our results showed that autistic children had difficulties in speed processing across all three areas—visual temporal, auditory temporal, and visuospatial processing—compared to the CG, suggesting speed challenges in integrating spatial, temporal, and physical information across modalities. These findings support hypotheses H1b, H2b, and H3b. Nonetheless, our findings contradicted hypotheses H1a and H2a, as autistic children outperformed the CG in accuracy on both visual and auditory temporal tasks, indicating a potential strength in temporal integration related to accuracy.

Significant differences in visual temporal processing tasks showed higher accuracy in the AG compared to the CG, indicating a heightened focus on detail or enhanced visual processing [123]. However, greater accuracy does not always equate to greater efficiency, as response times also affect overall performance [124,125]. Longer mean reaction times for correct mean responses suggest slower visual processing due to cognitive traits like integration impairments [12,126]. This aligns with research indicating autistic individuals process information more analytically [127,128]. For incorrect responses, autistic participants also showed longer mean reaction times, supporting the hypothesis of a more deliberative thinking process [129]. This trend was specific to autism, as no significant differences in incorrect mean reaction times were observed between the Developmental and Chronological CG. The AG’s accuracy reflects meticulous visual processing strategies, highlighting implications for understanding their decision-making processes, especially in accuracy-focused contexts [130,131]. These findings are contrary to our hypothesis H1a, but support H1b, as autistic children exhibited higher accuracy, but slower mean reaction times in visual temporal processing, compared to CG.

In auditory tasks, the AG achieved higher accuracy compared to the Chronological CG, and slower mean reaction time compared to both CG, despite characteristics like enhanced sensitivity to auditory pitch perception [132]. Nonetheless, it is important to highlight that their accuracy was similar to the developmentally matched CG, suggesting that auditory processing may function at a similar level in both autistic and non-autistic children [133]. Longer mean reaction times in autistic individuals for both correct and incorrect responses reflect slower processing speed, which is consistent with previous findings [134], favoring deliberation [129], possibly due to sensory overload [135]. These findings contradict our hypothesis H2a, but confirm hypothesis H2b, as autistic children exhibited higher accuracy compared to Chronological CG, but slower mean reaction times compared to CG in auditory temporal processing.

In visuospatial tasks, no significant differences in accuracy were found among AG, Developmental, and Chronological CG, suggesting comparable competencies across groups. However, autistic participants exhibited longer mean reaction times for both correct and incorrect responses. These findings suggest that while visuospatial abilities are preserved in autism, visual processing appears delayed—a pattern consistent with research showing slower executive processing in autistic individuals, particularly when responding to spatial cues in working memory tasks [136]. Also, our results are aligned with a meta-analysis that reported significantly longer mean reaction times in individuals with autism on time-based tasks, indicating slower processing speed in the autistic population [134], suggesting that neurotypicals tend to engage in more intuitive, faster, and automatic cognitive processing compared to autistic individuals [129]. Thus, hypothesis H3a is not supported, as there was no significant difference concerning visuospatial tasks accuracy, but H3b is confirmed as autistic children exhibited speed deficits in visuospatial processing compared to CG.

Comparing tasks revealed distinct patterns within the AG. Higher accuracy in visuospatial tasks compared to visual temporal tasks suggests a temporal disparity, marked by slower global processing in individuals with autism [137]. No accuracy differences between visual and auditory temporal tasks indicate modality-independent temporal processing [138]. Longer mean reaction times in auditory tasks highlight the complexity of auditory stimuli and possibly, heightened sensitivity to sounds and altered sensory processing in autism [28]. Visuospatial tasks showed mean response times comparable to visual temporal tasks, reinforcing the idea that visual processing can be a strength in autism, especially when individual abilities are correctly recognized and supported, as seen in autistic individuals who excel in artistic domains [17]. Therefore, our findings highlight intra-group variability across the three modalities within the autism group, which, when contrasted with previous between-group comparisons, contradicts one of our initial hypotheses (H1a and H2a) while supporting another (H1b, H2b, and H3b). Autistic children outperformed neurotypical peers in accuracy for visual and auditory temporal tasks, contradicting hypotheses H1a and H2a, which predicted accuracy deficits in those modalities, suggesting a strength in temporal integration related to accuracy in autism. However, regarding processing speed, our results support hypotheses H1b, H2b, and H3b, positing that speed processing deficits are observed across all three domains (visual and auditory temporal, and visuospatial), so autistic challenges likely involve broader difficulties in integrating spatial, temporal, and physical information, what could be most notably for auditory stimuli.

In chronologically and developmentally matched CG, results varied with modality, with significant differences in accuracy and mean reaction times. Similar patterns across the three groups suggest that AG performance may not be as unique as assumed, as both autistic and neurotypical children balance speed and accuracy in complex tasks, which is a common feature in decision-making processes [139]. For both Chronological and Developmental CG, visuospatial tasks showed the highest accuracy, followed by auditory and visual temporal tasks. Because the environment is constantly changing, visual input also shifts over time, relying on two key processes: separating moments to detect change and linking them to recognize patterns over time [140]. While frontoparietal regions support the retention of visual input, neural oscillations vary by type of temporal or spatial processing, as parietal areas show strong activity across frequencies for holding temporal details, while frontal and central regions are more engaged in theta and beta bands during spatial maintenance [141]. Although both spatial and temporal processes can jointly modulate the sensory responses [33], our results reflect stronger visuospatial processing and integration abilities compared to visual temporal processes, demonstrating efficient spatial integration. This trend, consistent across groups, suggests a more efficient visuospatial processing, potentially preserved in autism [90]. Concerning the mean reaction times, auditory tasks were slowest, followed by visuospatial and visual temporal tasks, suggesting faster, more direct processing in the visual modality. The greater auditory temporal accuracy alongside slower mean reaction times compared to visual temporal tasks suggests that the auditory system is more precise, but takes a longer processing speed, concerning temporal processing, than the visual system, which may be more attuned to spatial details, underlying that their timing mechanisms likely differ substantially [142]. Slower mean reaction times in auditory tasks, comparing to the other two tasks, for both the CG and AG (when analyzing data within the groups) may reflect the complexity of auditory stimuli, as a study on neural timing during image and sound perception found that brain responses to visual and auditory features began and peaked almost simultaneously, though peak activation occurred later for sound [143]. According to the study, this delay may reflect fundamental differences between modalities, as auditory input unfolds over time, with higher-level meaning processed later than basic sensory details. Although autistic children may show deficits in auditory processing [28], leading to the slowest mean reaction times in the auditory modality across groups, a slower processing compared to the visual modality is consistent in all groups, though our findings suggest it is more pronounced in autistic children.

To sum up, this study was based on the central idea that individuals with autism show behavioral differences associated with challenges in processing quantitative information through visual and auditory sensory processing [92], alongside difficulties in perceptual cognition involving the spatial and temporal dimensions of sensory experience [90,91]. Overall, our study suggests that the way autistic children process visual and auditory sensory input in relation to spatial and temporal perception follows a more complex and distinct pattern. Rather than reflecting a simple deficit, this sensory processing style, which considers spatial-temporal perception, appears to differ from that of neurotypicals—favoring a more analytical approach. Our study showed that autistic children outperformed neurotypicals in accuracy on both visual and auditory temporal tasks, suggesting a potential strength in temporal integration tied to accuracy, aligning with research showing that autistic individuals tend to process information more analytically [127,128]. Also, no significant differences in visuospatial accuracy between AG and CG indicate preserved spatial perception and processing abilities, consistent with findings that visual perception in autism is less impaired in tasks not requiring rapid temporal integration [123]. Nonetheless, the higher mean reaction times for autistic children across all modalities emphasize accuracy over speed, suggesting consistent temporal processing mechanisms involving detailed, stimulus-independent analysis [144]. Temporal and spatial processing rely on distinct neural networks, explaining observed differences [145], with temporal modality concerning processing speed emerging as a key vulnerability in autism. Interestingly, these findings converge with the Fitts’ Law reciprocal aiming task, a widely recognized paradigm for examining the tradeoff between accuracy and speed in motor performance, often used to investigate the interplay of action, perception, and imagination in autistic individuals [146].

### 4.1. Practical Implications for Interventions, Education, and Daily Life

We found that children with autism, compared to neurotypicals, achieved higher accuracy in temporal processing tasks (visual and auditory) but exhibited longer response times in all modalities (temporal visual and temporal auditory and visuospatial), suggesting an attentional and decision-making strategy prioritizing accuracy over speed [147]. This approach may reflect compensatory mechanisms enabling detailed sensory analysis, reducing errors at the expense of slower responses [148]—a pattern that should be taken into account in clinical, therapeutic, educational, and everyday interactions with autistic children.

Autism is recognized as a common, lifelong, and varied condition, shaped by a neurodiversity-informed perspective [3], that must be considered when thinking about interventions and educational strategies to support children’s health and development. Clinically, this means that assessments and treatments should include a gradual acclimatization of autistic children, avoid time-pressured and noisy environments, allow for calm, steady pacing, and use clear instructions and visual cues to support understanding and communication [149]. At school and home, creating structured, consistent routines with visual aids can lower stress and boost involvement [150]. Limiting overstimulation from noise, lighting, and textures and offering safe, quiet spaces can increase comfort and attention through a more relaxed educational experience [151]. The slower pace of autistic children in processing sensory input should be considered in sensory-heavy school environments by offering quiet, well-organized spaces that help create a more accessible and manageable setting [152]. Although our findings indicate that visuospatial accuracy remains intact, difficulties in combining sensory and timing information persist, highlighting the need for specialized strategies in clinical, therapeutic, and learning contexts [153]. Intervention and therapeutic programs play a key role in improving social interaction, reducing stress and anxiety, and enhancing the quality of life for autistic children [154,155,156]. Therefore, approaches aligned with our findings—emphasizing a slower temporal processing, particularly in visual and auditory temporal modalities—may lead to more effective outcomes.

Drawing on our findings, autistic children benefit from interventions that respect their longer processing times and avoid time pressure for task accuracy. Individuals with autism often display longer processing times, affecting cognitive and social functioning, and research suggests that interventions targeting processing speed may enhance social communication skills, offering a valuable therapeutic direction [157]. Early interventions for toddlers with autism have also demonstrated long-term benefits in cognition and communication, which may indirectly support improvements in processing speed [158]. Thus, the following therapeutic approaches may be particularly suitable. First, the Developmental, Individual-differences, Relationship-based (DIR^®^) model is a relationship-centered therapy that uses each child’s unique biological profile to guide personalized engagement, emphasizing—along with creative arts therapies—the value of supporting development by following the autistic child’s own initiative [159]. Second, Sensory Integration Occupational Therapy (SI-OT), based on Ayres theory, offers individualized interventions tailored to each child’s sensory profile, aiming to improve communication (both expressive and receptive), social interaction, and daily living skills by fostering sensory processing in a way that respects the child’s own pace [160]. Third, Pivotal Response Treatment (PRT) is a behavioral approach that adopts a more naturalistic approach, designed to enhance social communication in autistic individuals by boosting engagement through child-preferred, intrinsically motivating activities that encourage spontaneous interaction [161,162].

In educational and everyday contexts, our findings suggest practical accommodations that could enhance autistic children’s performance. For instance, slower processing in these children may hinder their ability to complete timed school tests [134], but their higher response accuracy found in this research indicates that, given enough time, they may show better performance. Therefore, unanswered or incorrectly answered questions may reflect time constraints rather than knowledge gaps. Offering extended time during assessments can be beneficial, and shifting the focus from one-time tests to cumulative work—such as portfolios or project-based learning (PBL)—may better reflect their true abilities. Also, a child who processes the world at a slower pace may require more time and effort to explore their surroundings, which can limit opportunities for simultaneous engagement in social interactions [163]. Therefore, allowing autistic children sufficient time to engage with their sensory surroundings before introducing social demands may help prevent overstimulation and support more adaptive behaviors afterward. Indeed, play-based activities can promote body awareness, balance, touch, and social participation [164], and may offer a more adaptable and less time-restricted form of interaction.

### 4.2. Future Research Implications Regarding the Digital Life

While our research design may not provide direct empirical evidence on the impact of digital environments, it is important to consider how sensory processing and perception affect autistic children’s experiences in a world that is increasingly digital [165]. Digital tools are often presented as adaptable solutions for education and therapy [166], supporting everyday functioning, learning, productivity, and leisure in autistic adolescents [167], with many features highly stimulating, rapidly changing, and multitasking demands that contrast with our findings emphasizing the benefits of slower-paced environments [168]. For young children still developing sensory and cognitive control, particularly those with autism, these conditions may be overwhelming and linked to negative developmental outcomes [169]. Indeed, some studies indicate that excessive screen use is associated with stronger autism-related symptoms [170], and that early audiovisual exposure in infancy may reinforce sensory pathways at the expense of social brain development in genetically susceptible children, explaining traits like atypical speech and face processing and underscoring the need for greater public awareness into the potential causal link between early screen exposure and autism [171]. The American Academy of Pediatrics (AAP) and the World Health Organization (WHO) advise no screen time for children under two and suggest a maximum of one hour daily for ages 2–5 [172], while other recommendations limit recreational screen use to two hours daily for individuals aged 5–17, not counting educational use [173]. Despite this, autistic children tend to exceed these limits, with average screen use reaching over three hours per day – more time than their neurotypical peers [174]. Our results therefore reinforce the importance of following screen-time guidelines and balancing digital exposure with offline and outdoor activities that provide calmer, slower-paced environments [175]. In this context, parent-focused programs to reduce screen time and increase social interaction have been well accepted by families, showing promise as practical approaches [176]. 

## 5. Conclusions

Recent studies on autism have advanced understanding and informed strategies to enhance the quality of life. This research compared sensory processing in visual, auditory, and visuospatial modalities across children with autism and neurotypicals, using Cedrus SuperLab tasks in familiar settings. In our study, the autistic sample comprised predominantly boys with higher cognitive functioning. The children with autism showed higher accuracy in visual and auditory temporal tasks but required longer mean response times, reflecting precision-focused strategies. Conversely, neurotypicals demonstrated faster, more efficient sensory integration. Similar accuracy in visuospatial tasks across groups highlights visuospatial processing as a potential autism strength. Temporal processing in autism prioritizes accuracy over speed, especially in visual and auditory modalities. Thinking interventions and educational strategies that take into account the child’s full context—at home, school, and in daily life—and align with these processing tendencies found in our results can support healthy development, strengthen social interactions, lower stress and anxiety, and improve overall quality of life. Adoption of clinical and educational approaches that reflect each child’s traits, including the slower and more deliberate cognitive strategies of autistic children, should be considered. In today’s fast-paced digital world, autistic children are especially sensitive to overstimulating environments, with excessive screen time linked to poorer developmental outcomes. Our findings contextualize the need to limit exposure, encourage slower-paced engagement, and balance screen use with offline activities. 

## 6. Limitations and Future Research

While this study provides valuable insights into temporal and spatial processing in children with autism, several limitations should be considered. First, sample characteristics, though carefully controlled, may limit the generalizability of the findings to broader autism populations, as ensuring unbiased estimates in a target group depends on maintaining both internal and external validity [177]. Many studies rely on homogeneous datasets, limiting generalizability to the broader autism community, highlighting the need for future research to include culturally, ethnically, and socioeconomically diverse data—an issue cross-cultural studies can address by examining symptom expression across populations [178]. Although contextual approaches consider identity and social ecology, autism research has rarely integrated cultural factors, and doing so could enhance relevance, strengthen construct validity, and provide a clearer understanding of autism outcomes [179]. Culture directly impacts how autism is expressed and interpreted—for instance, while collectivist cultures may intensify social challenges for autistic children due to expectations around reciprocity, individualistic societies often value direct communication, as seen in East Asian cultures like Japan, where greater social distance is favored, potentially leading Western evaluators to misread such behavior as social disengagement [180]. Regarding gender, important differences in the autism population must also be considered, such as camouflaging—strategies to appear less autistic in social settings—which is more common in autistic females and may delay their screening, recognition of their needs, and access to support [181]. Additionally, autistic females tend to exhibit stronger social interaction and communication skills than males, reflecting a gender-related pattern also observed in nonautistic populations [182]. Concerning age, literature suggests that autistic individuals may not show the usual age-related gains in key skills and brain maturation during chronological development, such as executive function, social cognition, communication, emotional recognition, and self-awareness [95]. Our sample focused on a specific age range (4 to 6 years), was recruited from Portuguese institutions, and was predominantly male-factors that should be taken into account when interpreting the results. Although this study did not focus on contextual or cultural aspects of autism, these elements are essential in autism research, and future research should include more diverse populations and account for greater variability in cognitive and sensory profiles. 

Second, the reliance on computer-based tasks, despite their precision, may not fully capture sensory integration challenges faced by autistic children in dynamic environments. Technology-based approaches involve the intentional use of electronic tools or platforms to aid daily life, education, and interventions in autistic adolescents [167]. Technology-based interventions can reach large numbers of children with minimal reliance on mental health professionals, though outcomes and data from these tools may not always transfer effectively to real-world settings [183]. For instance, in contrast to computer-based image-watching tasks (videos, pictures, web pages), face-to-face interaction is a social task that is much more perceptually and cognitively demanding for autistic people [109]. Autistic traits may not always result in noticeably atypical social attention during real-life interactions, as many autistic individuals may behave in masking—using learned behaviors to appear more neurotypical—while limited opportunities to practice social attention in screen-based settings may make differences between autistic and non-autistic individuals more apparent [108]. On the other hand, computer-based interventions can broaden assessment and treatment options for autistic children—for example, eye-tracking technology, which responds to a child’s gaze, shows promise in evaluating and supporting attention in very young children with early signs of autism, offering a practical solution for toddlers and preschoolers [184]. Therefore, it is important to consider the strengths and limitations of digital and non-digital assessments and interventions with autistic children when interpreting data and evaluating the generalizability of results.

Third, while our study does not directly assess the effects of digital environments on autistic children, our findings underscore the importance of considering how sensory and perceptual processing shape their experiences in a fast-paced, tech-driven world [165,166,167]. Many digital tools promote overstimulation and rapid multitasking, which may overwhelm autistic children and be linked to negative developmental outcomes [169], especially when screen exposure exceeds recommended limits [174]. Also, our findings highlight the need to allow a slower pace in visual and auditory tasks for autistic children—a strategy that should guide the design of more inclusive, flexible, and supportive environments [19,185]. Future research and interventions should adopt evidence-based, inclusive, and adaptable design principles that reflect the unique processing profiles of autistic children, while also examining the impact of screen exposure—particularly considering their slower sensory processing patterns highlighted in this study.

Lastly, children and adolescents with autism frequently experience a wide range of somatic and psychiatric co-morbidities—including ADHD, anxiety, depression, epilepsy, intellectual disability, sleep disorders, sensory impairments, and gastrointestinal issues—which contribute to the condition’s clinical variability [186]. The elevated prevalence of co-morbidities in autism may stem in part from shared underlying risk factors, such as genetic predispositions or environmental influences, rather than being solely a consequence of autism itself [187]. Prenatal information regarding maternal medication use (e.g., antidepressants, ADHD stimulants) or substance use during pregnancy was also not systematically collected, which limits our ability to rule out these potential influences on child development. Therefore, the study did not account for potential comorbidities or differences in developmental trajectories, which could influence performance in sensory processing tasks. Furthermore, information regarding participants’ use of anti-seizure medication or other prescribed drugs was not systematically collected, which limits our ability to account for potential pharmacological influences on child development. Future studies should address these limitations by incorporating more diverse samples and multidimensional assessments to enhance ecological validity [188,189].

## Figures and Tables

**Table 1:** Hypotheses.

Processing Modality	Hypotheses	Hypotheses Description
Visual Temporal Processing	H1a	Autistic individuals exhibit deficits in visual temporal processing compared to neurotypicals, regarding processing accuracy.
	H1b	Autistic individuals exhibit deficits in visual temporal processing compared to neurotypicals, regarding processing speed.
Auditory Temporal Processing	H2a	Autistic individuals exhibit deficits in auditory temporal processing compared to neurotypicals, regarding processing accuracy.
	H2b	Autistic individuals exhibit deficits in auditory temporal processing compared to neurotypicals, regarding processing speed.
Visuospatial Processing	H3a	Autistic individuals exhibit deficits in visuospatial processing compared to neurotypicals, regarding processing accuracy.
	H3b	Autistic individuals exhibit deficits in visuospatial processing compared to neurotypicals, regarding processing speed.

**Table 2:** Sample Description.

Variable	Group	Mean	Standard Deviation
Age	AG	62.9	6.946
(months)	Chronological	67.29	5.861
	Developmental	67.5	6.661
Raven’s	AG	15.57	5.219
Score	Chronological	16.21	4.048
	Developmental	15.57	5.043
Variable	Group	Percentage	
Gender		Girls	Boys
	AG	18.76	81.25
	Chronological	53.57	46.43
	Developmental	40.63	59.38

**Table 3:** Descriptive statistics of the group × sensory modality factorial design with repeated measures in sensory modality.

Variable	Group	Mean	Standard Deviation	*n*
Proportion Correct (Visual)	AG Group	0.61810	0.082347	29
	Chronological CG	0.30583	0.087268	30
	Developmental CG	0.46250	0.151764	28
	Total	0.46034	0.169496	87
Proportion Correct (Visuospatial)	AG	0.69304	0.079176	29
	Chronological CG	0.69935	0.096655	30
	Developmental CG	0.67017	0.067725	28
	Total	0.68785	0.082391	87
Proportion Correct (Auditory)	AG	0.57780	0.114975	29
	Chronological CG	0.42114	0.094340	30
	Developmental CG	0.51742	0.125136	28
	Total	0.50435	0.128497	87
Mean Reaction Time (Correct, Visual)	AG	5548.0194	2844.92692	29
	Chronological CG	2341.1035	451.06680	30
	Developmental CG	2760.4483	929.84217	28
	Total	3545.0370	2243.67825	87
Mean Reaction Time (Correct, Visuospatial)	AG	5588.7866	2126.07044	29
	Chronological CG	3213.3321	295.97822	30
	Developmental CG	3563.2797	820.63016	28
	Total	4117.7771	1681.60370	87
Mean Reaction Time (Correct, Auditory)	AG	6734.9140	2631.65505	29
	Chronological CG	4329.3552	193.62329	30
	Developmental CG	4688.6469	722.15939	28
	Total	5246.8422	1890.24199	87
Mean Reaction Time (Incorrect, Visual)	AG	5226.3212	2734.05984	29
	Chronological CG	2230.2407	333.84586	30
	Developmental CG	2504.5807	737.81964	28
	Total	3317.2275	2120.94882	87
Mean Reaction Time (Incorrect, Visuospatial)	AG	5525.9358	1794.49644	29
	Chronological CG	3496.6668	715.97360	30
	Developmental CG	3671.2680	755.63844	28
	Total	4229.2833	1502.04175	87
Mean Reaction Time (Incorrect, Auditory)	AG	6371.9631	2415.56475	29
	Chronological CG	4243.5340	170.86549	30
	Developmental CG	4660.3571	780.77953	28
	Total	5087.1603	1721.97657	87

**Table 4:** Univariate tests for the main effects of the sensory modality factor and the interaction effects of sensory modality × group.

Source	Variable	Sum of Squares (Type III)	df	Mean Squares	F	*p*	Partial η^2^
Sensory Modality	Proportion of Correct Responses	2.487	2	1.243	181.303	<0.001	0.683
	Mean Reaction Time (Correct) (1)	130,278,892.78	2	65,139,446.39	102.872	<0.001	0.550
	Mean Reaction Time (Incorrect)	136,451,420.15	2	68,225,710.08	118.123	<0.001	0.584
Sensory Modality × Group	Proportion of Correct Responses	0.769	4	0.192	28.016	<0.001	0.400
	Mean Reaction Time (Correct) (1)	7,993,158.408	3.688	2,167,492.208	3.156	0.019	0.070
	Mean Reaction Time (Incorrect)	11,521,246.651	4	2,880,311.663	4.987	<0.001	0.106
Error	Proportion of Correct Responses	1.152	168	0.007			
	Mean Reaction Time (Correct) (1)	106,379,444.80	154.885	686,827.308			
	Mean Reaction Time (Incorrect)	97,034,014.544	168	577,583.420			

**Table 5:** S Univariate Tests for the Group Factor, df (2, 84).

Variable	Type III Sum of Squares	Error Sum of Squares	Mean Square	Error Mean Square	F	*p*	Partial η^2^
Proportion Correct	1.052	1.502	0.526	0.018	29.421	<0.001	0.412
Mean Reaction Time (Correct)	363,169,009.48	505,859,606.48	181,584,504.74	6,022,138.172	30.153	<0.001	0.418
Mean Reaction Time (Incorrect)	296,011,427.00	431,332,379.79	148,005,713.50	5,134,909.283	28.823	<0.001	0.407

**Table 6:** Estimated Marginal Means for the Group Factor.

Measure	Group	Mean	Standard Error	95% Confidence Interval [Inferior, Superior Limit]
Proportion Correct	AG	0.630	0.014	[0.601, 0.658]
	Chronological	0.475	0.014	[0.447, 0.503]
	Developmental	0.550	0.015	[0.521, 0.579]
Mean Reaction Time (Correct)	AG	5957.24	263.10	[5434.04, 6480.44]
	Chronological	3294.60	258.67	[2780.19, 3809.00]
	Developmental	3670.79	267.75	[3138.33, 4203.25]
Mean Reaction Time (Incorrect)	AG	5708.07	242.94	[5224.95, 6191.20]
	Chronological	3323.48	238.86	[2848.48, 3798.48]
	Developmental	3612.07	247.24	[3120.40, 4103.74]

**Table 7:** Estimated Marginal Means for the Sensory Modality Factor.

Measure	Sensory Modality	Mean	Standard Error	95% Confidence Interval [Inferior, Superior Limit]
Proportion Correct	Visual	0.462	0.012	[0.438, 0.486]
	Visuospatial	0.688	0.009	[0.670, 0.705]
	Auditory	0.505	0.012	[0.482, 0.529]
Mean Reaction Time (Correct)	Visual	3549.857	187.188	[3177.612, 3922.102]
	Visuospatial	4121.799	142.022	[3839.372, 4404.227]
	Auditory	5250.972	169.213	[4914.473, 5587.471]
Mean Reaction Time (Incorrect)	Visual	3320.381	176.404	[2969.582, 3671.180]
	Visuospatial	4231.290	128.432	[3975.889, 4486.692]
	Auditory	5091.951	157.302	[4779.139, 5404.764]

**Table 8:** Estimated Simple Means.

Measure	Group	Sensory Modality	Mean	Standard Error	95% Confidence Interval [Inferior, Superior Limit]
Proportion Correct	AG	Visual	0.618	0.021	[0.577, 0.659]
		Visuospatial	0.693	0.015	[0.663, 0.723]
		Auditory	0.578	0.021	[0.536, 0.619]
	Chronological	Visual	0.306	0.020	[0.266, 0.346]
		Visuospatial	0.699	0.015	[0.669, 0.729]
		Auditory	0.421	0.020	[0.381, 0.462]
	Developmental	Visual	0.462	0.021	[0.421, 0.504]
		Visuospatial	0.670	0.016	[0.639, 0.701]
		Auditory	0.517	0.021	[0.475, 0.559]
Mean Reaction Time (Correct)	AG	Visual	5548.019	324.091	[4903.528, 6192.511]
		Visuospatial	5588.787	245.892	[5099.802, 6077.771]
		Auditory	6734.914	292.970	[6152.311, 7317.517]
	Chronological	Visual	2341.104	318.644	[1707.445, 2974.762]
		Visuospatial	3213.332	241.760	[2732.567, 3694.097]
		Auditory	4329.355	288.046	[3756.545, 4902.166]
	Developmental	Visual	2760.448	329.828	[2104.549, 3416.347]
		Visuospatial	3563.280	250.245	[3065.640, 4060.919]
		Auditory	4688.647	298.156	[4095.732, 5281.562]
Mean Reaction Time (Incorrect)	AG	Visual	5226.321	305.419	[4618.961, 5833.681]
		Visuospatial	5525.936	222.363	[5083.743, 5968.129]
		Auditory	6371.963	272.347	[5830.371, 6913.555]
	Chronological	Visual	2230.241	300.286	[1633.089, 2827.392]
		Visuospatial	3496.667	218.626	[3061.906, 3931.428]
		Auditory	4243.534	267.769	[3711.045, 4776.023]
	Developmental	Visual	2504.581	310.826	[1886.470, 3122.691]
		Visuospatial	3671.268	226.299	[3221.248, 4121.288]
		Auditory	4660.357	277.168	[4109.179, 5211.535]

## Data Availability

The original data for the auditory stimuli presented in the study are openly available in the Open Science Framework (OSF).
